# Clinical Consequences of Unreconstructed Pelvic Defect Caused by Osteosarcoma with Subsequent Progressive Scoliosis in a Pediatric Patient—Case Report

**DOI:** 10.3390/children11050607

**Published:** 2024-05-19

**Authors:** Sławomir Zacha, Katarzyna Kotrych, Wojciech Zacha, Jowita Biernawska, Arkadiusz Ali, Dawid Ciechanowicz, Paweł Ziętek, Daniel Kotrych

**Affiliations:** 1 Department of Children Orthopedics and Musculoskeletal Oncology, Pomeranian Medical University of Szczecin, 71-252 Szczecin, Poland; slawomir.zacha@pum.edu.pl (S.Z.);; 2 Department of Anesthesiology and Intensive Therapy, Pomeranian Medical University of Szczecin, 71-252 Szczecin, Poland; 3 Department of Orthopedics, Traumatology and Musculoskeletal Oncology, Pomeranian Medical University of Szczecin, 71-252 Szczecin, Poland

**Keywords:** adolescent idiopathic scoliosis, osteosarcoma, surgical treatment, hemipelvectomy, 3D printed implant

## Abstract

Osteosarcoma is the most common primary malignant bone tumor in children and adolescents. The standard and most effective treatment is wide resection of the tumor combined with neoadjuvant chemotherapy. Adolescent idiopathic scoliosis (AIS) is a genetically determined three-dimensional spinal deformity, which occurs in teenage patients and is mostly progressive. The basic management strategy is surgical treatment when the curve exceeds 50 degrees. However, the indications are different in oncologic patients. The aim of this study was to describe a case of adolescent scoliosis with osteosarcoma of the pelvis. The authors conducted a scoping review using PubMed and Embase to analyze the state of knowledge. The presented paper is the first report of pelvis osteosarcoma coexisting with adolescent idiopathic scoliosis. Treatment for this complex case finished with very good results, with no recurrence observed during the nine-year follow-up.

## 1. Introduction

Osteosarcoma is the most common primary malignant bone tumor in children and adolescents [1]. Its first peak of incidence occurs in patients between 15 and 25 years of age and the second peak occurs in patients above 50 years of age. The incidence rate amounts to around 0.5 to 1 per million population per year and is more common in males [2]. The most common sites of occurrence are the femur, tibia and humerus, followed by the pelvis and skull [3]. Tumors located around the pelvis and a lack of proper reconstruction of postoperative bone defects cause secondary axial skeleton deformities. Symptoms deriving from a pelvic bone sarcoma may appear late as a dull, intermittent pain after exercise, which then becomes continuous and worsens at night. Systemic symptoms, such as fever or weight loss, are rare. The basic diagnostic method is magnetic resonance imaging supported by computed tomography, and finally, biopsy [4]. The standard and most effective treatment is wide resection of the tumor combined with neoadjuvant chemotherapy [5,6,7].

Adolescent idiopathic scoliosis (AIS) is a genetically determined, three-dimensional spinal deformity, which occurs in teenage patients and is mostly progressive [8]. Conservative treatment of AIS aims to stop or slow down the progression of spinal deformation. Management alternatives depend on age, bone maturity, type and severity of curvature. Also, the rate of progression has a major impact on the choice of treatment [9]. Treatments include scoliosis-specific physiotherapy and bracing, which can significantly slow AIS deterioration. When the progressive spinal curvature exceeds approximately 45–50 degrees, the basic management strategy is surgical treatment [8,10]. The indications for surgical treatment are different in oncologic patients, as the problem of cancer must be solved first.

The aim of this study was to describe a case of adolescent scoliosis with osteosarcoma of the pelvis.

## 2. Materials and Methods

To analyze the state of knowledge about the coexistence of scoliosis and osteosarcoma of the pelvis in the pediatric population, we conducted a scoping review. The methodological framework for conducting a scoping review was based on rules proposed by Arksey and O’Malley [11]:

1. Identifying the research question: “what are the clinical consequences of unreconstructed pelvic defect caused by osteosarcoma with subsequent progressive scoliosis in a pediatric patient?”. The authors analyzed the state of knowledge about the consequences of the coexistence of idiopathic scoliosis with osteosarcoma of the pelvis in the pediatric population.
2. Identifying relevant studies: Two authors analyzed data in the PubMed and Embase databases using the keywords, “pelvic osteosarcoma”, “idiopathic scoliosis” and “children”. A manual search for references was then performed using the eligible publications describing the clinical course of these comorbidities in children. Studies were searched for in English, with no restrictions on publication time.
3. Study selection: This involved post hoc inclusion and exclusion criteria. Study selection was performed by the first and last authors.
4. Charting the data: A data-charting form was developed and used to extract data from each study. A ‘narrative review’ method was used to extract process-oriented information from each study.
5. Collating, summarizing, and reporting results.

## 3. Results

### 3.1. Scoping Review

What are the clinical consequences of unreconstructed pelvic defects caused by osteosarcoma with subsequent progressive scoliosis in a pediatric patient? There are numerous scientific reports on the occurrence of scoliosis of various etiologies in children and separate reports describing cases of osteosarcoma. Searching both databases with predefined keywords, we found no articles. When we removed “idiopathic” and “children”, we found one article describing a retrospective review plus two representative case reports. However, the mean age of patients was 47 years old [12,13].

### 3.2. Patient Presentation

**Figure 1:**
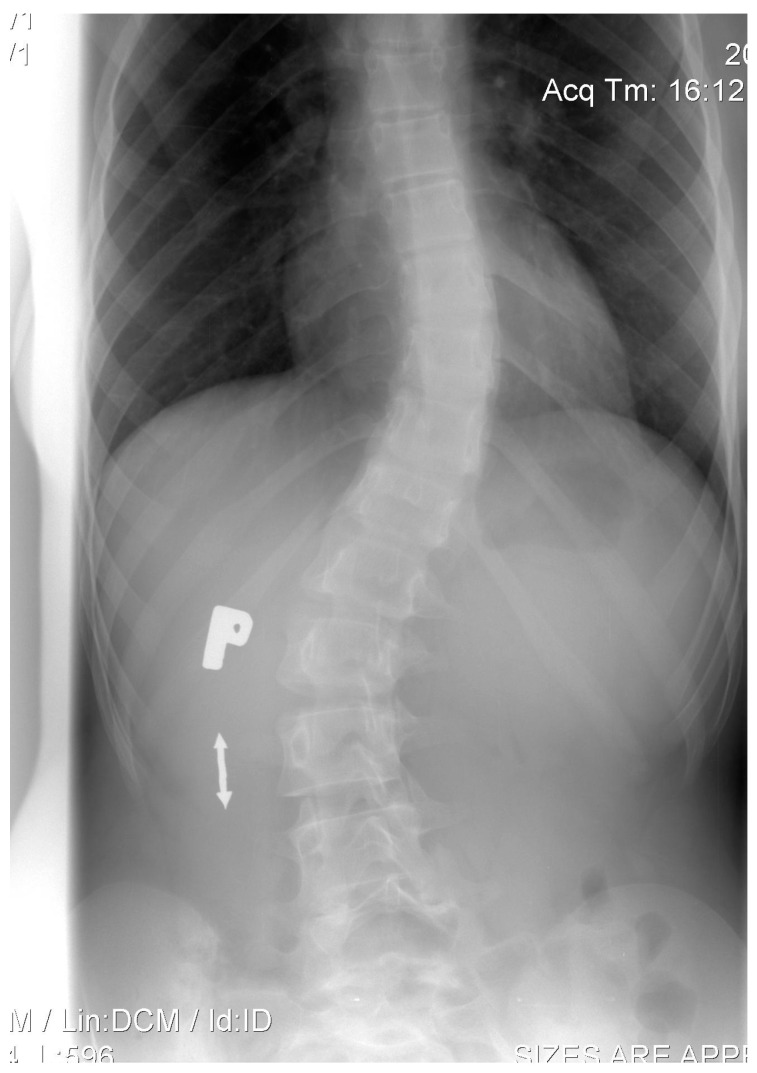
Lenke 1B- at the age of 14.

We present a case of a 12-year-old female patient who, in 2008, was diagnosed with AIS (Lenke 1B-, Cobb angle 32° of thoracic curve). She was treated conservatively with only physiotherapy for 2 years. Despite the implemented treatment, progression of up to 40° of the thoracic curve was observed (Figure 1).

At that time, she started to complain of acute pain in the right hip and iliac bone, which did not subside after taking painkillers and was exacerbated during the night. Due to the presence of coexisting scoliosis, the patient was not diagnosed properly at first, as the pain was attributed to her spine condition. After consulting another orthopedic surgeon during scoliosis treatment, additional blood tests and pelvis X-rays were ordered. Results showed elevated alkaline phosphatase (ALP) and inflammation markers. By that time, the patient was having trouble walking without crutches. A physical examination revealed pain around the right sacroiliac joint without swelling and with unrestricted spine and hip range of motion.

**Figure 2:**
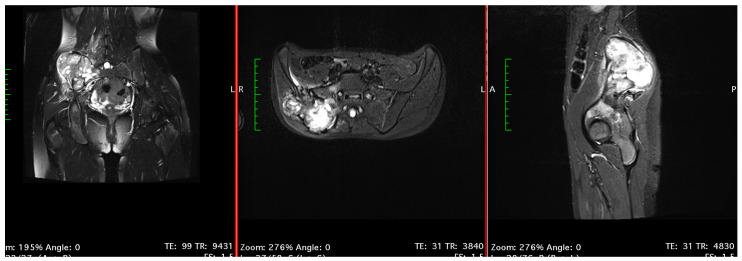
Osteosarcoma of the right ilium in MRI view.

The patient was admitted to a medical center in Opole, Poland, in December 2012, where additional imaging was conducted. Magnetic resonance imaging (MRI) showed a tumor localized in the right iliac bone, measuring 8.6 × 7.4 × 12.5 cm in size (Figure 2). Multiple metastases in the lungs were also documented using chest computed tomography (CT). The girl was urgently transferred to the University Department of Pediatric Oncology and Bone Marrow Transplantation in Wroclaw, Poland.

### 3.3. Patient Management & Outcomes

#### 3.3.1. Pelvis Osteosarcoma

**Figure 3:**
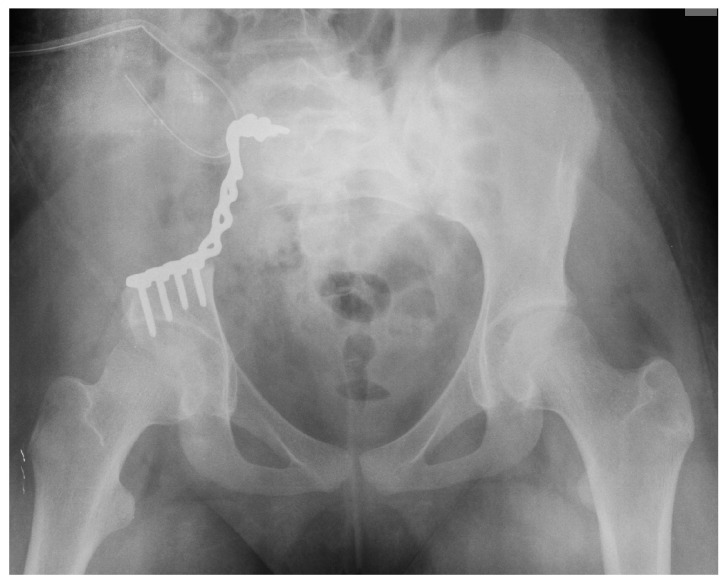
Postoperative X-ray.

Chemotherapy was administered according to protocol after the biopsy revealed the diagnosis of osteosarcoma. Due to the full remission of metastases after 10 months, hemipelvectomy with illiosacral stabilization using a plate was performed in The Department of Pediatric Surgery of Lower Silesian Specialized Hospital in Wroclaw in October 2013 (Figure 3). The histopathological test confirmed the diagnosis of osteosarcoma (high-grade, G3; mixed form; pleomorphic-like).

**Figure 4:**
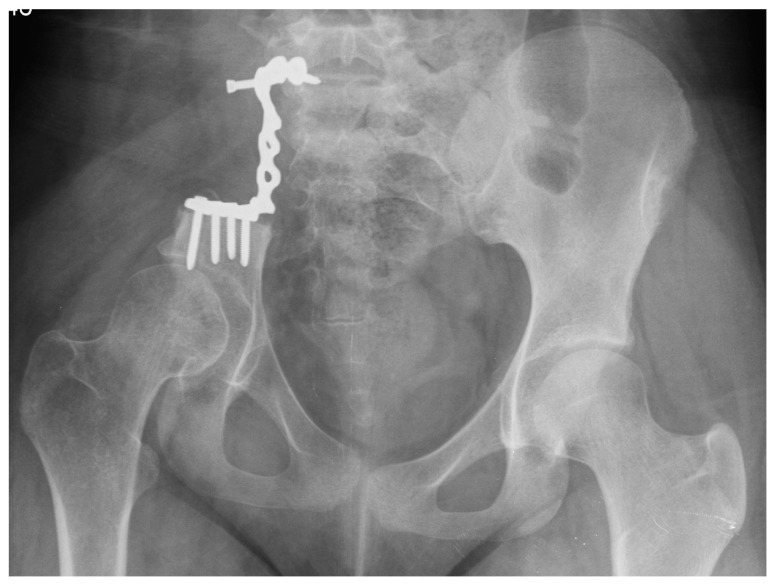
Breakage and destabilization of the plate.

Postoperatively, paresis of the right sciatic nerve was noticed. The resection margins were confirmed to be clear (R0). Unfortunately, the method of reconstruction used proved to be inadequate. Six weeks after surgery, the pelvis happened to break and the pelvic ring was destabilized, causing biomechanical insufficiency. For this reason, the decision was made to proceed with the next stage of surgical treatment using a similar internal fixation. It was complicated by a screw perforating into the L4/L5 intervertebral space, damaging the L4 root on the right side (Figure 4). The patient presented with severe symptoms, such as unbearable neuropathic pain, sciatic nerve palsy and complete walking disability. The patient was dismissed from the surgical department and completed complementary chemotherapy within nine months. Throughout all the periods spent under oncologic treatment, the patient was unable to walk, presented with persistent radiculopathy and a neurologic deficit, and developed depression.

**Figure 5:**
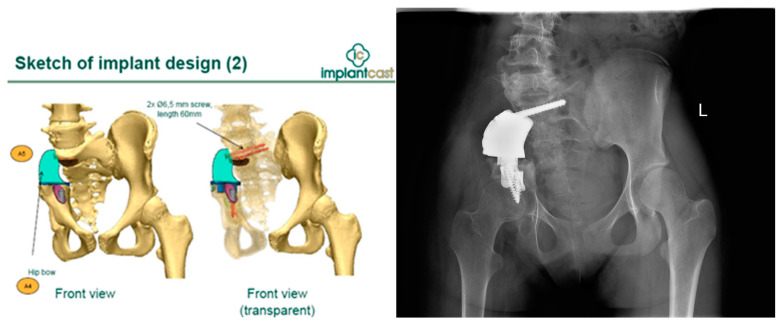
MUTARS 3D-printed implant design and postoperative X-ray view.

Finally, systemic therapy had been completed. Due to the failure of the last surgical treatment, the patient was referred to another medical center specializing in cancers of the musculoskeletal system in children—Department of Orthopedics, Traumatology and Musculoskeletal Oncology in Szczecin, Poland. She was qualified for revision surgery and pelvic defect reconstruction by means of custom-made MUTARS 3D-printed endoprosthesis (Implantcast^®^, Hamburg, Germany). The implant design was based on 0.6 mm CT scans. The idea was not only to restore the defect, but also to support the sacrum to restore proper trunk balance (Figure 5).

The surgery was performed in April 2015. The patient was placed in a floppy lateral position on the healthy side. An incision was made in a postoperative scar, and the broken plate was exposed and removed. The 3D-printed prosthesis was implanted and attached to the remaining iliac and sciatic bone, and to the sacrum at the level of S1 and S2 (Figure 5). There were no postoperative complications. Within the first seven days following 3D reconstruction, the pain significantly diminished to VAS 4, and the patient was able to walk alone with crutch support. Within the next six months, the sciatic nerve recovered from complete damage to 50% of its functional level. For another two years, the patient functioned well without pain and did not require pharmacological treatment.

She was in a constant rehabilitation program and under outpatient orthopedic control. Within that time, we observed substantial osseointegration of the pelvic implant, confirmed by SPECT-CT bone scintigraphy.

#### 3.3.2. Spine Deformity

**Figure 6:**
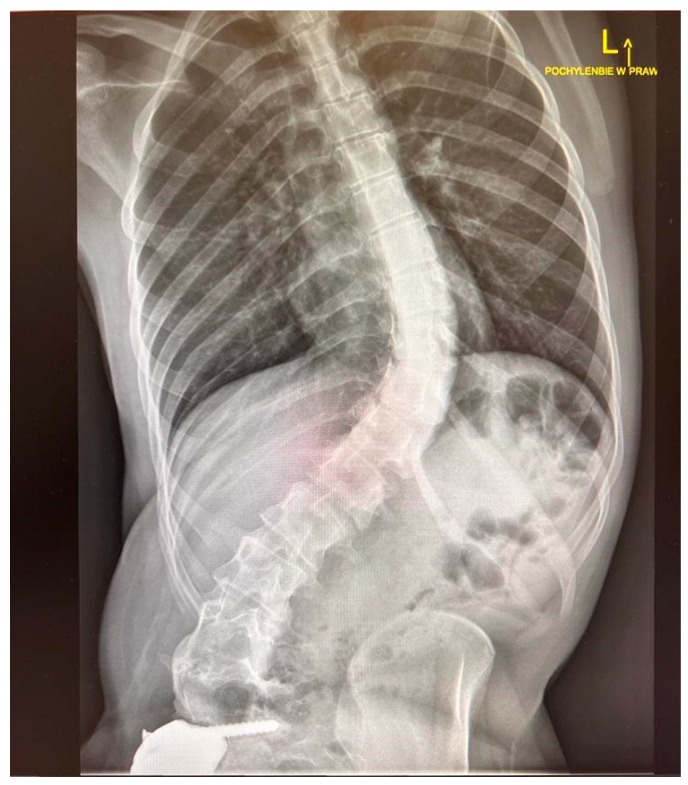
Progression and transformation of curve(Cobb 68°)—before surgery.

What was first a Cobb angle of 32° progressed to a Cobb angle of 68°, which required surgical intervention (Figure 6). Additionally, the curve morphology changed from double-curve Lenke 1B to a longitudinal thoracolumbar left curve due to the oblique orientation of the L4 upper endplate. At the age of 21, due to an increasing functional disorder and persistent back pain, the decision for surgical reduction of the spine was made in January 2021. The long period between the treatment of a malignant pelvic tumor and the correction of scoliosis was caused by the lack of consent for the next procedure by the patient and her family. Magnetic resonance imaging of the spine performed before spine surgery did not show any abnormalities in the neural structures.

**Figure 7:**
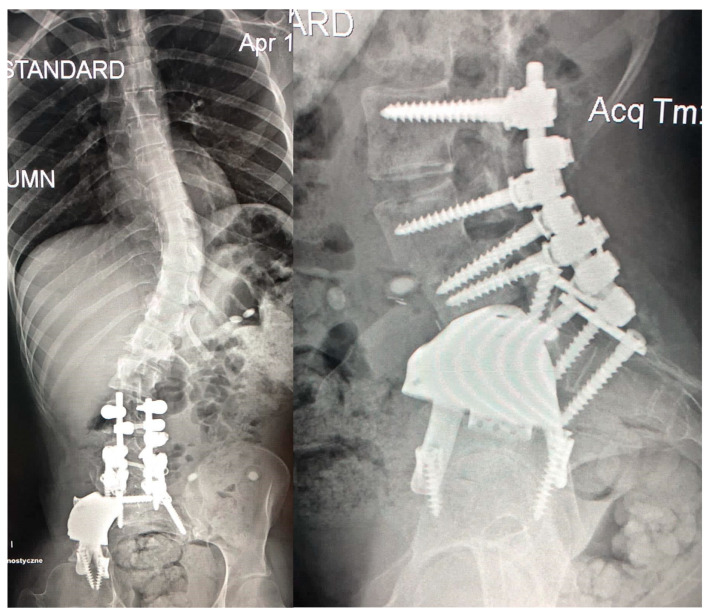
Surgical treatment stage one with L4 osteotomy and posterior stabilization VL3-VS2—directly after surgery.

The aim of the first stage was to reduce the deformity of the lower lumbar part and to achieve a proper position of the L4 upper endplate. The procedure was performed in the Independent Public Specialized Health Care Center in Szczecin–Zdroje, Poland. The procedure involved the transpedicular fusion of L3-S2 from a posterior approach, and a lateral wedge osteotomy of L4 and L5 from an anterior approach (Medicrea^®^, Rillieux-la-Pape, France) (Figure 7). During the osteotomy and ossified tissue removal, the external iliac vein was damaged and repaired by a vessel surgeon, which significantly prolonged the operation to up to nine hours. The postoperative recovery period passed without complication. The patient was discharged from the hospital eleven days postoperation.

**Figure 8:**
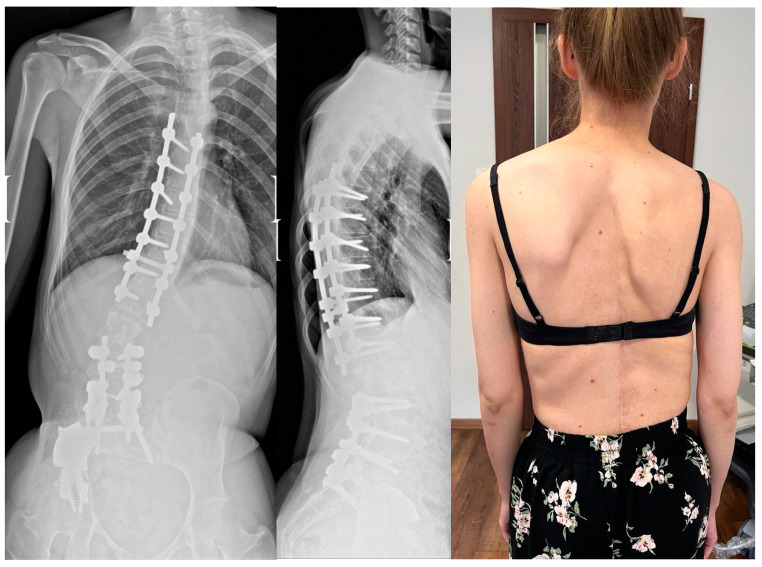
Surgical treatment stage two—transpedicular selective reduction of VTh7-VL1; last follow-up.

In the second stage of surgery in December 2021, transpedicular selective reduction of Th7-L1 was performed from a posterior approach (Medicrea^®^, France) (Figure 8). In both stages of surgical treatment of spinal deformities, the screws were inserted using the free-hand technique. The decision to perform a selective spondylodesis leaving two free segments, L1/L2 and L2/L3, was made together with the patient and her family as a proposal to preserve mobility in the above-mentioned levels. They were informed about the risk of degenerative changes and instability, and the possible need for another stage of surgical treatment. They did not agree to fuse the entire operated spine. There were no postoperative complications. The patient was discharged from the hospital nine days postoperation.

#### 3.3.3. Clinical Outcomes

The clinical outcomes include back pain relief and further recovery from sciatic nerve palsy. After two years of rehabilitation, the patient walks with the help of crutches for longer distances and with no assistance in her flat. The persistent trunk shift visible in the X-rays was a result of right hip instability and compensation for body balance. We did not observe any deterioration in scoliosis or spinal imbalance during the follow-up. The function of the urinary bladder and anal sphincters remained intact throughout the entire treatment.

The patient is disease-free eight years after the first tumor resection and pain-free three years after scoliosis reduction. She can walk with the help of crutches for around 1000 m without stopping, and without them for around 50 m. Currently, she can perform daily activities by herself. During the last follow-up, a permanent decrease in superficial sensation on the lateral side of the right lower extremity was observed. Weakened muscle strength of the right hip abductors (Lovett 3) and right foot extensors (Lovett 3) was found according to the Lovett scale. Finally, the patient regained the ability to live independently.

## 4. Discussion

This presented paper is the first report of pelvis osteosarcoma coexisting with adolescent idiopathic scoliosis. Treatment of this complex case finished with fair results considering the circumstances, with no recurrence of the malignant tumor during the nine-year follow-up after pelvic reconstruction. It is necessary to point out that surgical interventions resulted in the stiffening of two extensive sections of the spine with two movable segments: L1/L2 and L2/L3. The risk for the patient is the occurrence of instability and degenerative changes in adjacent parts of the spine in the future, and the need for further surgical procedures. Moreover, the weakening of the abductor muscles of the operated hip joint and partial damage to the sciatic nerve significantly impaired the patient’s walking ability.

Hemipelvectomy due to osteosarcoma is a lifesaving surgery and long-term survival is poor. The complication rate of hemipelvectomy is between 40 and 60% [14,15,16,17,18]. Currently, the treatment method using custom-made 3D-printed prostheses is considered a golden standard [19].

As shown by Beck et al., both internal and external hemipelvectomies were performed using different kinds of stabilization, but 3D-printed prostheses were not used.

The initial treatment of this presented case, using a plate for stabilizing the sacrum to the iliac bone, was not efficient enough. It caused an implant failure and the necessity of additional treatment. Partial sciatic nerve palsy, which occurred in our case, is often unavoidable according to the literature [20].

Sacroiliac instability is a common complication after hemipelvectomy with different stabilization implants. This biomechanical impact on spine function may cause iatrogenic deformities, which require surgical intervention [21].

There was a single case report of scoliosis operative reduction after external hemipelvectomy and one case report of scoliosis that occurred after revision surgery for pelvic reconstruction and spinopelvic fixation, in which the authors decided against operative reduction of scoliosis [12,13]. This presented case report is the first one with scoliosis reduction in a patient who previously underwent 3D reconstruction of the pelvis after hemipelvectomy. What makes this case unique is the conversion and progression of preexisting idiopathic scoliosis into an iatrogenic deformity caused by unilateral spinal fusion with the steel plate used in the first and second pelvic surgeries. Even if the scoliosis was considered idiopathic, its etiology was potentially tumor-related from the beginning.

There are not many articles describing this topic in the available literature. Using the keywords “scoliosis” and “pelvis tumors”, one can find 38 items in the PubMed database. However, most of them are not relevant to our research. The vast majority of them describe the development of secondary scoliosis after pelvic ring reconstruction, postoperative development of desmoid tumors after surgical correction of adult spinal deformities, the adult population with tumors in the pelvis or degenerative scoliosis in adults. One of them describes a biomechanical study.

There are only two articles relevant to our topic. Wang et al. showed a rare case of osteoblastoma combined with a severe scoliosis deformity in a 14-year-old girl. However, the authors believed that the scoliosis deformity, pelvic obliquity and spinal imbalance were caused by this benign tumor. The patient underwent tumor excision and scoliosis correction at the same time. The patient had full neurological recovery with no aggravation of scoliosis or spinal imbalance during the follow-up [22].

Jackson and Gokaslan described the results of treatment for 13 patients who required spinal–pelvic fixation secondary to instability caused by lumbosacral neoplasms to prevent secondary scoliosis [23].

We deem our results satisfactory, all things considered. Although the radiological results of spine deformity correction are not excellent, we achieved proper cosmetic and functional results that are acceptable for both us and the patient.

## 5. Limitations

We acknowledge that our study had its limitations due to the incomplete radiological documentation before the final treatment. This is because the patient was treated in various medical centers in Poland initially, and we were unable to obtain all the data we needed.

## Data Availability

The original contributions presented in the study are included in the article, further inquiries can be directed to the corresponding author.
